# Current Symptomatic and Disease-Modifying Treatments in Multiple System Atrophy

**DOI:** 10.3390/ijms21082775

**Published:** 2020-04-16

**Authors:** Lisa Mészáros, Alana Hoffmann, Jeanette Wihan, Jürgen Winkler

**Affiliations:** Department of Molecular Neurology, University Hospital Erlangen, Friedrich-Alexander-University Erlangen-Nürnberg, 91054 Erlangen, Germany; lisa.meszaros@uk-erlangen.de (L.M.); alana.hoffmann@uk-erlangen.de (A.H.); jeanette.wihan@uk-erlangen.de (J.W.)

**Keywords:** multiple system atrophy, parkinsonian disorders, drug development, disease-modifying therapy

## Abstract

Multiple system atrophy (MSA) is a rare, severe, and rapidly progressive neurodegenerative disorder categorized as an atypical parkinsonian syndrome. With a mean life expectancy of 6–9 years after diagnosis, MSA is clinically characterized by parkinsonism, cerebellar ataxia, autonomic failure, and poor l-Dopa responsiveness. Aside from limited symptomatic treatment, there is currently no disease-modifying therapy available. Consequently, distinct pharmacological targets have been explored and investigated in clinical studies based on MSA-related symptoms and pathomechanisms. Parkinsonism, cerebellar ataxia, and autonomic failure are the most important symptoms targeted by symptomatic treatments in current clinical trials. The most prominent pathological hallmark is oligodendroglial cytoplasmic inclusions containing alpha-synuclein, thus classifying MSA as synucleinopathy. Additionally, myelin and neuronal loss accompanied by micro- and astrogliosis are further distinctive features of MSA-related neuropathology present in numerous brain regions. Besides summarizing current symptomatic treatment strategies in MSA, this review critically reflects upon potential cellular targets and disease-modifying approaches for MSA such as (I) targeting α-syn pathology, (II) intervening neuroinflammation, and (III) neuronal loss. Although these single compound trials are aiming to interfere with distinct pathogenetic steps in MSA, a combined approach may be necessary to slow down the rapid progression of the oligodendroglial associated synucleinopathy.

## 1. Introduction

Multiple system atrophy (MSA), an atypical parkinsonian disorder, is a sporadic and rare neurodegenerative disorder affecting 1.9–4.9 of 100,000 people with an average onset in the sixth decade of life and a mean survival of 6–9 years [1]. The fast progressive disease course is clinically characterized by two major motor phenotypes: the parkinsonian (MSA-P) and cerebellar subtype of MSA (MSA-C) mainly presenting parkinsonism and cerebellar ataxia, respectively [1,2]. Additionally, a profound autonomic failure such as orthostatic hypotension, urinary incontinence, constipation, and erectile dysfunction is a mandatory symptom in MSA. Based upon these different clinical phenotypes the diagnosis of MSA is clinically categorized either as “possible” or “probable” according to present consensus criteria [2]. In case patients show a sporadic, progressive adult-onset disease with parkinsonism or cerebellar ataxia and at least one additional symptom indicative for dysautonomia combined with other clinical or neuroimaging features, “possible” MSA is diagnosed. For the category of “probable” MSA, the patients must present a sporadic, adult-onset, and progressive disease course including cerebellar or parkinsonian symptoms with poor l-Dopa responsiveness in combination with autonomic symptoms. [2]. Since there is currently no valid clinical, biochemical, or imaging biomarker established, a “definite” MSA diagnosis requires the neuropathological presence of oligodendroglial cytoplasmic inclusions (GCI) containing alpha-synuclein (α-syn) aggregates. This is the most prominent structural hallmark of MSA [2,3,4]. α-Syn is a 14-kDa protein encoded by the *SNCA/PARK1* gene and physiologically involved in neurotransmitter synthesis and release [5]. However, its accumulation is closely associated with a variety of neurodegenerative diseases, classified as α-synucleinopathies. While the intracellular expression of α-syn is well described for neurons, the origin of oligodendroglial GCIs is still debated. One finding suggests an endogenous expression of α-syn in oligodendrocytes, strengthened by the identification of α-syn transcripts in isolated nuclei of oligodendroglial lineage cells derived from rodents and humans [6]. Alternatively, an uptake of α-syn from neighboring cells or the extracellular environment combined with the involvement of specific oligodendroglial proteins e.g., tubulin polymerization-promoting protein (TPPP/p25α) have been proposed [7,8,9,10]. Even though the origin of α-syn in MSA has not yet been clarified, its accumulation may interfere with important oligodendroglial functions. Despite an unaltered number of oligodendrocytes in white matter regions in the fore- and hindbrain, myelin formation is severely impaired resulting in severe myelin loss [11,12,13,14,15,16,17,18]. Reduced myelination is accompanied by pronounced neuronal loss in distinct brain regions, including the motor cortex, dorsolateral putamen, globus pallidus, cerebellum, and substantia nigra correlating with GCI density and disease progression [3,13,15,19]. Furthermore, neuroinflammation is an important pathological feature of MSA consisting of micro- and astrogliosis driving an increased release of inflammatory cytokines such as tumor necrosis factor alpha (TNFα), interferons, and interleukins (IL), predominantly in the white matter of the central nervous system (CNS) [20,21,22,23].

Although considered as a sporadic disease, several familial cases of MSA were observed suggesting a genetic predisposition for the disease. Indeed, mutations in the *COQ2* gene, encoding the enzyme para-hydroxybenzoate-polyprenyl transferase have been identified in a Japanese MSA patient cohort and were proposed as a genetic risk factor [24]. Located at the inner mitochondrial membrane, coenzyme Q10 is an essential cofactor for the mitochondrial respiratory chain. Thus, mutations in the *COQ2* gene may result in mitochondrial dysfunction, a crucial pathogenic event frequently associated with neurodegenerative diseases [25]. However, conflicting results have emerged since mutations in the *COQ2* gene were not detected in non-Asian patient cohorts [24,26]. Further genetic studies linked specific SNCA polymorphisms [27,28,29] and α-syn mutations such as A53E and G51D with an increased risk of developing MSA [30,31]. Besides a genetic predisposition, several environmental factors including the exposure to metal dusts and fumes, plastic monomers, and pesticides have been discussed as potential risk factors. However, how and to which extent these factors contribute to MSA pathology needs further investigation [32,33]. So far, aging remains the sole, well-accepted risk factor for developing MSA.

Due to the limited knowledge regarding the precise underlying pathogenesis and molecular targets triggering MSA, there is currently no disease-modifying therapy available for MSA patients. However, the rapid and severe disease progression as well as the orphan disease status makes MSA particularly interesting for advanced drug development and accelerated approval. This review provides an overview of the neuropathology of MSA, summarizes current symptomatic treatment strategies, and more importantly reflects on potential disease-modifying approaches for MSA.

## 2. Neuropathology of MSA

Neuropathological prerequisite of “definite” MSA are proteinaceous aggregates predominantly detected in the cytoplasm of oligodendrocytes visualized by Gallyas silver staining. GCIs or so-called Papp–Lantos bodies are agryophilic, granulated, and loosely packed with a diameter of 5–20 µm. They appear in various morphologies having half-moon, triangular, or oval shape [3]. Less frequently, other inclusions have been found in MSA patients including protein aggregates in the nuclei of oligodendrocytes and neurons, in the cytoplasm of neurons, as well as in astroglia [34,35,36]. Similar to neuronal Lewy bodies in Parkinson’s disease (PD) and dementia with Lewy bodies (DLB) patients, α-syn is the predominant component within glial protein aggregates in MSA [37,38]. Furthermore, a variety of other proteins has been identified in GCIs including ubiquitin, tau, tubulin, heat shock proteins, and p25α [35].

Examination of post-mortem brain tissue of MSA patients revealed brain atrophy due to neurodegeneration and myelin loss, in particular, in the striatonigral and olivopontocerebellar tracts of MSA-P and MSA-C patients, respectively [39]. However, neuronal loss has also been described in autonomic nuclei such as the Onuf’s nucleus, nucleus ambiguous, and pontomedullary brain stem or cortical regions [15,40]. Clinicopathological correlations linked GCI density and neurodegeneration in distinct brain regions to corresponding symptoms [15,40,41,42]. In 2004, Ozawa and colleagues observed a link between disease duration, neuronal loss, and GCI density, whereas, other studies reported a high variability among patients [43,44,45]. Besides neuronal pathology, degeneration of white matter associated with severe myelin loss is another important macroscopical hallmark in post-mortem studies of MSA patients [3,14,39]. Interestingly, oligodendroglial numbers were not reduced in white matter regions [12,17,18]. Due to early oligodendroglial pathology and progressive α-syn aggregation, Wenning and colleagues suggested MSA to be a primary oligodendrogliopathy followed by a secondary neurodegeneration [14,46]. Furthermore, a regional specific inflammatory response was described in MSA patients predominantly composed of micro- and astroglia [18]. Increased numbers of microglial cells and its activation has been shown in white matter regions depending on the disease stage and α-syn levels [23,47,48]. Activation of astrocytes has also been detected in a variety of brain regions including the olivopontocerebellar, striatonigral, and corticospinal tract as well as autonomic systems [3,21,43,45]. Recently, the presence of T cells has been demonstrated indicating a broad spectrum of inflammatory cell populations in MSA post-mortem brain tissue [49].

## 3. Current Symptomatic Treatment of MSA

Symptomatic treatment targeting the above described clinical symptoms ultimately aims to improve quality of life. Ongoing clinical trials are summarized in Table 1.

MSA-P patients show parkinsonian symptoms including bradykinesia, rigidity, postural instability, tremor, and freezing of gait. Furthermore, these parkinsonian symptoms are also present in 50%–74% of MSA-C patients [56,57,58]. Treatment with l-Dopa is considered as gold standard for the treatment of PD and counteracts bradykinesia and rigidity in MSA patients as well. However, the majority of MSA patients show a poor or fast declining l-Dopa responsiveness [57,59]. The lack of l-Dopa response is considered as an initial “red flag” to distinguish sporadic PD from MSA-P. Although dopamine agonists show no beneficial effect in non-l-Dopa-responsive MSA patients [60], at least 22% of the l-Dopa responders show diminished parkinsonian symptoms [57]. Treatment with monoaminooxidase B (MAO-B) inhibitors such as rasagiline is feasible, however, no benefit has been shown in MSA patients [57]. Currently, the safety and tolerability of the MAO-B inhibitor and glutamatergic signaling modulator safinamide is being tested as add-on therapy to l-Dopa in a placebo-controlled and double-blinded phase 2 trial [50]. Furthermore, the *N*-methyl-d-aspartate (NMDA) receptor inhibitor amantadine may be an alternative for an add-on therapy to ameliorate parkinsonian symptoms. However, the efficacy of amantadine is questionable due to the lack of informative studies. Thus, more comprehensive studies are still needed [60,61,62,63].

The most frequent cerebellar signs in MSA-C patients are gait ataxia, limb ataxia, and ataxic dysarthria. These cerebellar symptoms are also occurring in 41–64% of MSA-P cases [56,57,58]. Physiotherapy especially consisting of gait and balance training has shown to alleviate gait impairment [64]. Nevertheless, there is no efficacious therapy for MSA-C-related ataxia available so far. However, cerebellar NMDA receptors play a considerable role in motor learning and coordination, thus, may be a feasible target for treating ataxia [65]. Accordingly, the efficacy of the NMDA receptor modulator Tllsh2910 is currently being tested in a double-blinded, randomized, cross-over phase 3 study in MSA patients [51].

Dystonia, a further common motor symptom in MSA, is treated by local injections of Botulinum toxin A and may result in functional improvement [66]. However, adverse side effects of this therapy were described such as provoking severe dysphagia [67].

Autonomic failure, including urinary symptoms, orthostatic hypotension, constipation, and erectile dysfunction are mandatory characteristics for the diagnosis of “possible” or “probable” MSA. Although being present in 99% of all MSA cases, the minority of MSA patients receive pharmacological therapy addressing dysautonomia [56,57,58].

Urinary symptoms are one of the most common dysautonomic features occurring in 71–83% of MSA patients [56,57,58]. Anticholinergic drugs such as oxybutynine, trospium chloride, and tolterodine reduce urinary incontinence [56,68,69]. Furthermore, the application of intranasal desmopressin is an alternative option in order to diminish nocturnal polyuria and abolish sleep fragmentation [70]. If pharmacological measures are not beneficial, intermittent self-catheterization should be considered [69].

Orthostatic hypotension is a common symptom in about 75–78% of MSA patients [56,58]. Therefore, the usage of the sympathomimetic ephedrine might be feasible, although no decisive clinical evidence is available at present [71]. Furthermore, treatment with midodrine, a peripheral α1-selective adrenoceptor agonist, is an effective and well-tolerated substance to overcome orthostatic hypotension [72]. A randomized, double-blinded, 2-arm parallel phase 1 study is currently evaluating the effects of midodrine and droxidopa on blood pressure in MSA patients [52]. l-threo-dihydroxy-phenylserine (l-threo-DOPS, droxidopa) is a precursor of norepinephrine, which increases blood pressure by its conversion to norepinephrine [38]. An ongoing placebo-controlled, double-blinded, randomized phase 4 study is currently investigating the optimal dosage of droxidopa in patients suffering from MSA [53]. An alternative approach to increase levels of norepinephrine is via blockade of the norepinephrine transporter (NET). The NET inhibitor atomexetine is more efficacious in improving orthostatic hypotension than midodrine in MSA patients [73]. Moreover, pyridostigmine is able to potentiate the effect of atomexetine on blood pressure and diminishes orthostatic symptoms by enhancing cholinergic neurotransmission [74]. Currently, an open-label phase 2 study aims to determine the optimal dose of atomoxetine in MSA patients which will be followed by a randomized, double-blinded, placebo-controlled crossover trial [54]. Additionally, the norepinephrine reuptake inhibitor (NRI) ampreloxetine (TD-9855) is currently being tested in a randomized, double-blinded, placebo-controlled phase 3 study on tolerability, safety, and efficacy in patients with primary autonomic failure including those with MSA [55]. Furthermore, the mineralocorticoid fludrocortisone might ameliorate hypotension by targeting renal mineralocorticoid receptors and, thus, increase water retention and intravascular volume. While there are no convincing clinical studies in MSA patients, fludrocortisone showed efficacy in patients suffering from orthostatic hypotension [75]. Additionally, non-pharmacologic treatment such as the usage of elastic stockings must be considered [76]. One third of MSA patients suffer from constipation and respond to the osmotically acting laxative macrogol [56]. In addition, erectile dysfunction being present in 84–94% of male MSA patients is effectively treated using the phosphodiesterase 5 inhibitor sildenafil [56,58,59].

Conclusively, symptomatic treatments are available for MSA patients. However, the majority of treatments are poorly validated in MSA. The current trial activity for symptomatic treatment in MSA will help to improve both the clinical care and the quality of life of MSA patients.

## 4. Potential Disease-Modifying Targets in MSA

Based upon the major pathologic hallmarks of MSA, the following disease-modifying targets and current clinical trials are summarized in Table 2.

### 4.1. Targeting alpha-Synuclein Aggregation

The most prevalent neurodegenerative diseases such as Alzheimer’s disease (AD), PD, Huntington’s disease, amyotrophic lateral sclerosis, and MSA are proteinopathies associated with the presence of distinct abnormal protein aggregates in different neural populations. In particular, α-syn is a neuronal monomeric and natively unfolded protein, which pathologically converts into misfolded oligomers and subsequently forming cell inclusions [5]. These α-syn aggregates define MSA and PD as synucleinopathies and play an important role in the underlying neurodegenerative pathomechanisms [89]. Since α-syn accumulation occurs in numerous brain regions of MSA patients and its respective models that correlate with gliosis and neuronal loss, as well as disease severity and duration, the inhibition or prevention of α-syn accumulation constitutes an obvious target to interfere with the disease course [3,13,15,19].

One potential approach originating in the field of AD is active immunization strategies against the specific disease-related protein in order to attenuate protein accumulation by increasing the clearance of the respective protein. For instance, Affitope AFF1, PD01A, and PD03A are vaccines consisting of different short synthetic α-syn peptide fragments, which aim to promote immune system-mediated production of antibodies against α-syn. Six-time monthly vaccination of 4–5 months old transgenic mice overexpressing human α-syn under a myelin basic protein (MBP) promoter line 1 (MBP1-hα-syn mice) were treated with Affitope AFF1 resulting in the production of specific blood–brain barrier-permeable anti-α-syn antibodies. This active immunization reduced α-syn accumulation, demyelination, and neurodegeneration [90]. A randomized, placebo-controlled phase 1 study assessed the safety, tolerability, and immunogenicity of Affitope PD01A and PD03A in early MSA patients [77]. After four subcutaneous injections of PD01A or PD03A with adjuvants every 4th week, patients received a final immunization 9 months after the first injection. Both Affitope PD01A and PD03A were considered as safe. Additionally, this study indicates that PD01A, but not PD03A, induces an immune response against the peptide itself and α-syn [91]. Furthermore, PD01A was detected in cerebrospinal fluid and resulted in a tendency to reduce oligomeric α-syn levels in plasma as well as cerebrospinal fluid after treatment with PD01A at week 26 [92]. Recently, Affiris announced a phase 2 study with PD01A in early PD patients for 2020 [93]. In this regard, a randomized, placebo-controlled and double-blind phase 1 study recently started investigating the safety and tolerability of the synthetic α-syn vaccine UB-312 in PD patients, possibly also applicable for MSA patients [94,95]. However, it remains unclear whether high-weighted macromolecular antibodies are able to cross the blood–brain barrier sufficiently and, thus, represent an effective therapeutic approach for CNS diseases.

Besides active immunization, levels of α-syn in the CNS may also be lowered via passive immunization using specific antibodies against α-syn epitopes. Weekly injections of specific anti-α-syn antibodies over a period of 6 months indicated reduced cortical and hippocampal α-syn aggregates and reversed learning deficits in 12 months old transgenic mice. These mice express human α-syn under the platelet-derived growth factor receptor β (PDGFβ) promoter (line D) representing a model of DLB [96]. Furthermore, injection of CD5-D5 targeting oligomeric species of α-syn resulted in a reduced α-syn accumulation in MBP1-hα-syn mice. Moreover, combining this approach with the anti-inflammatory compound lenalidomide showed stronger effects and partially ameliorated behavioral deficits in this mouse line [97]. While there are currently no active studies in MSA, a few clinical trials investigate passive immunization against α-syn in PD, such as the antibodies PRX002, MEDI134, and BIIB054, which may be also beneficial for MSA [94,98,99].

The polyphenol epigallocatechin gallate (EGCG) showed positive effects on the transformation of fibrillary toxic to smaller non-toxic α-syn oligomers and probably acts by directly converting the structure of specific toxic α-syn species into a non-toxic conformation [100,101]. Additionally, EGCG rescued the loss of dopaminergic neurons in 1-methyl-4-phenyl-1,2,3,6 tetrahydropyridine (MPTP)-lesioned mice as a model for PD, presumably due to its radical scavenging activity and ferric ion chelating property [100,102]. A randomized, double-blind, and placebo-controlled trial (PROMESA) investigated the safety and efficacy of orally administered EGCG in MSA [78]. Generally, EGCG was well-tolerated, albeit hepatotoxicity was observed in two out of the 47 EGCG-treated patients. Unfortunately, after 48 weeks of treatment and a consecutive wash-out period of 4 weeks no significant changes were observed in motor scores based upon the Unified Multiple System Atrophy Rating Scale (UMSARS) compared to baseline [103].

Initially, the blood–brain barrier permeable and orally available small molecule anle138b showed inhibitory effects on α-syn accumulation by blocking oligomer formation in prion-infected mice and different PD mouse models such as rotenone- or MPTP-lesioned mice as well as the [A30P]-α-syn transgenic mice. Additionally, anle138b diminished neuronal degeneration and disease progression in these mouse models [104]. Anle138b was investigated in a MSA mouse model overexpressing human α-syn under the proteolipid protein promoter (PLP-hα-syn mice) [105]. In fact, the oligomer modulator resulted in a reduction of oligomeric α-syn levels and GCIs as well as microgliosis in the substantia nigra, accompanied by reduced loss of dopaminergic neurons and improved motor functioning. These promising findings encourage further investigations in humans. Indeed, a phase 1 trial is currently under way in order to test the safety, tolerability, and blood levels of orally administered anle138b [106].

Recently, the orally bioavailable small molecule inhibitor of α-syn PBT434 received the first orphan drug designation for MSA. PBT434 presumably acts via binding of iron and therefore abolishes the iron-mediated α-syn accumulation. An improved motor function in conjunction with a reduction of α-syn accumulation and neuronal loss has been described in the substantia nigra in a toxic MSA model using mice lesioned with MPTP and 6-hydroxydopamine. Similar effects were also observed in a transgenic mouse model for familiar PD expressing the human A53T mutant form of α-syn (hA53T α-syn) under the control of the PDGFβ-promoter, and additionally in the PLP-hα-syn mice [107,108]. A first randomized, double-blind, placebo-controlled phase 1 trial in healthy individuals indicated a good tolerance of a single dose PBT434 (300 mg) with infrequent and mild, but no serious adverse effects [109].

In addition, the chelating agent and antioxidant NBMI (Iriminix) is currently being tested in a phase 2 study regarding efficacy and safety in patients suffering from progressive supranuclear palsy (PSP) or MSA. Besides investigating improvement of motor and non-motor symptoms as well as quality of life, the brain iron level will be evaluated using MRI imaging [79].

It is also worthwhile to note that RNA interference might be a pre-translational strategy to lower α-syn levels. In particular, the leucine rich repeat kinase 2 (LRRK2) antisense oligonucleotide BIIB094 has been previously shown to decrease the formation of α-syn inclusions in mice vaccinated with α-syn pre-formed fibrils [110]. This approach is currently being studied in a phase 1 trial in PD patients regarding safety, tolerability, and pharmacokinetics [111].

Notably, FTY20-Mitoxy, a derivate of the sphingosine-1-phosphate receptor modulator fingolimod clinically approved for multiple sclerosis (MS), reduced insoluble α-syn accumulation and prevented motor impairments in a transgenic MSA mouse model overexpressing α-syn under a 2′-3′-cyclic nucleotide 3′-phosphodiesterase (CNP) promoter (CNP-hα-syn mice). Additionally, FTY-Mitoxy increased glial cell line-derived neurotrophic factor (GDNF) at both mRNA and protein levels in the frontal cortex, and, moreover, reduced microglial activation in the cerebellum of CNP-hα-syn mice [112]. In addition, FTY720-Mitoxy has potent anti-inflammatory activity and stimulates the production of multiple neurotrophins as well as protective neuronal microRNAs [113,114,115].

In general, strategies of lowering α-syn levels may be a very promising target, as α-syn accumulation occurs already in an early stage of MSA. However, the efficacy of protein lowering strategies in patients with age-dependent proteinopathies such as synucleinopathies is still awaiting a compelling positive clinical outcome. Finally, lowering physiological soluble α-syn might cause adverse effects too [116].

### 4.2. Targeting Neuroinflammation

Neuroinflammation is a well-known pathological feature of MSA consisting of activation of microglia and astrocytes with an increased release of pro-inflammatory cytokines. Particular interest is related to the specific regional distribution of microgliosis in α-syn-overloaded white matter regions correlating with neurodegeneration [23,47].

Inhibition of myeloperoxidase (MPO), an enzyme involved in the synthesis of reactive oxygen species by phagocytic cells such as microglia, represents an attractive target for reducing neuroinflammation in MSA [117]. MPO inhibition resulted in a reduction of microgliosis and rescue of neurons in the striatum, substantia nigra, and cerebellar cortex of PLP-hα-syn mice combined with an acute lesion of 3-nitropropionic acid (3-NP) [118]. Based on these preclinical findings the blood–brain barrier-permeable, irreversible MPO inhibitor verdiperstat (BHV-3241) is currently being tested in a randomized, double-blinded, placebo-controlled phase 3 study in MSA. After oral treatment with 300 mg or placebo twice daily the efficacy of verdiperstat will be measured at week 48 by the Clinical Global Impression of Change (CGI-C) score for tracking a change of disease severity. Additionally, the impact on quality of life will be investigated by a change in the MSA-Quality of Life (MSA-QoL) scale from baseline to week 48 [80].

In order to reduce neuroinflammation and slow down disease progression, the inhibition of microglia- and astroglia-driven expression of TNFα is an additional promising target in MSA. As mentioned above, the combined treatment of the anti-α-syn-antibody CD5-D5 and lenalidomide, an anti-inflammatory drug reducing the expression of pro-inflammatory cytokines such as TNFα, counteracts behavioral deficits in MBP1-hα-syn mice [97]. Furthermore, inhibition of TNFα production and release by applying thalidomide preserved striatal dopaminergic neurons in a PD mouse model using MPTP-lesioned mice [119,120]. The CD20 antibody rituximab, which inhibits B-cell activation, is already approved for autoimmune diseases such as MS or rheumatoid arthritis. In a rodent spinal cord injury model rituximab reduced the expression of TNFα in neuronal and glial cells [121]. However, it remains unclear whether rituximab affects the production of naturally occurring autoantibodies possibly playing a crucial role in blocking pathological proteins such as α-syn [122]. An ongoing phase 2 study evaluating the efficacy of rituximab in MSA-C patients will shed light on the effect of B-cell inhibition in the context of oligodendroglial α-syn aggregation [81].

In conclusion, the inhibition of MPO peroxidase as well as the reduction of TNFα-dependent neuroinflammation are promising disease-modifying targets and clinically being tested in MSA at present.

### 4.3. Targeting Neuronal Loss

Numerous efforts have been undertaken to explore neuroprotective approaches to prevent neuronal loss, one of the major pathological and general features in neurodegenerative diseases such as MSA.

One potential strategy to delay neurodegeneration is the systemic application of autologous human bone marrow-derived mesenchymal stem cells (MSCs). Injection of MSCs into the tail vein of a double lesioned MSA-P mouse model using MPTP and 3-NP resulted in an improved survival of neurons in the substantia nigra and the striatum, ameliorated motor functions, and reduced microglial and astroglial activation [123]. These neuroprotective and immunoregulatory effects may be related to the increased MSC-driven release of neuro- and immunomodulators including vascular endothelial growth factor (VEGF), brain-derived neurotrophic factor (BDNF), and anti-inflammatory IL-6 as well as IL-10 [124,125]. Moreover, MSCs improved the phagocytic clearance of α-syn by polarization of microglia into a M2 phenotype [126]. In the last decade, three clinical trials provided evidence for the safety and tolerability of MSCs in patients suffering from MSA or spinocerebellar ataxia [127,128,129]. However, it remains unclear whether MSCs are able to substantially cross the blood–brain barrier under both healthy and pathological conditions [130]. Thus, three follow-up studies are currently investigating the safety and tolerability of intravenous, intracarotid, or intranasal administration of MSCs in MSA [82,83,84]. Additionally, a phase 1 trial will be started by mid-2020 to investigate the safety, tolerability, and pharmacokinetics of intrathecal injection of BIIB101 in MSA patients [85].

Another approach targeting neurodegenerative processes is the usage of insulin. Insulin resistance has been described in neurodegenerative diseases such as AD [131] and MSA [132]. Insulin resistance may play a crucial role in the dynamic interplay of neurodegenerative processes and metabolic disorders [133]. Treatment with the Glucagon-like peptide analogue exendin-4 in PLP-hα-syn mice ameliorated insulin resistance and decreased monomeric a-syn levels in the striatum. Additionally, increased survival of nigral dopaminergic neurons was observed, however, without improving motor performance [132]. Recently, a double-blinded, placebo-controlled phase 2 study evaluated the safety and efficacy after 4 weeks of daily intranasal insulin treatment in PD patients [86]. Despite the small number of enrolled subjects and the short period of treatment this study suggests the tolerability of intranasal insulin and improvement of motor function, reflected by a decreased Hoehn and Yahr stage of 21% and a reduction of the Unified Parkinson Disease Scale-Motor Score by 19% [134]. To confirm these results and to determine the disease-modifying efficacy of insulin in PD or MSA, further clinical studies with larger patient cohorts and long-term treatment are needed.

Another possible target for decreasing neuronal degeneration is the inhibition of the mechanistic target of rapamycin (mTOR) receptor. Rapamycin is an allosteric mTOR receptor modulator and inhibits distinct mTOR actions. This macrolide attenuated the decrease of neurite length in the human cell line SH-SY5Y. Furthermore, motor function was ameliorated in the transgenic hA53T α-syn PD mouse model [135], presumably by blocking the translation of the gene *RTP801* and thereby activating the survival-driving kinase Akt [136]. Additionally, there is some suggestive evidence that rapamycin preserves neurons by accelerating autophagy of α-syn [137]. Recently, a randomized, placebo-controlled phase 2 study started investigating the efficacy of the orally administered mTOR receptor inhibitor sirolimus to delay disease progression in MSA patients [87,138].

As oxidative stress is increased in MSA and associated with cognitive deterioration, antioxidants might have a beneficial effect on disease progression. Indeed, elevation of uric acid in serum levels positively correlates with motor and cognitive functions in PD and MSA [139,140,141]. Thus, treatment with the antioxidant and uric acid prodrug inosine 5′-monophosphate may be a possible treatment strategy. A phase 2 trial evaluated the safety, tolerability, and increase of serum uric acid in MSA patients treated with inosine 5′-monophosphate, however conclusive results are pending [88].

Gene therapy might constitute another feasible approach to diminish oxidative stress, glutamate-related excitotoxicity, and subsequently neuronal loss. Thus, lentiviral injection of the nuclear-related factor 2 (*NRF2*), the glutamate dehydrogenase 2 (*GDH2*), and the excitatory amino acid transporter 2 (*EAAT2*) genes in 3-NP-lesioned as well as in PLP-hα-syn mice revealed enhanced motor function [142]. Due to oxidative stress the transcription factor NRF2, which is involved in the upregulation of phase-II detoxification enzymes [143], elevates the expression of anti-oxidative and anti-inflammatory factors [144]. GDH2 and EAAT2 reduced glutamate levels and consequently neuronal damage [145,146]. Although these studies demonstrated improved motor function upon combined expression, further investigations are needed to evaluate effects on disease progression and to elucidate the underlying neuroprotective mechanisms.

Remarkably, mutations in the gene *COQ2*, which is required for the synthesis of coenzyme Q10, is associated with cerebellar ataxia in MSA-patients [147]. Coenzyme Q10 insufficient neurons derived from induced pluripotent stem cells from MSA patients showed increased oxidative stress and subsequently apoptosis [148]. This raises the question whether supplementation of coenzyme Q10 might be a well-tailored substitution in MSA patients carrying *COQ2* mutations. A first case report in a *COQ2* deficient patient provided a first hint that high-dose treatment with ubiquinol, the reduced form of coenzyme Q10, might be efficacious [149]. Currently, a randomized, double-blind, placebo-controlled phase 1 study is investigating safety and pharmacokinetics of the reduced form of coenzyme Q10 (MSA-01) in healthy male subjects [150].

Conclusively, the majority of the past clinical trials addressed neuronal loss in the context of neurodegenerative diseases, albeit all of them failed to slow or halt disease progression. As neuronal loss may occur as a consequence of oligodendroglial dysfunction at a more advanced stage in MSA, the question raises whether targeting neurodegeneration is a timely appropriate target to delay disease progression.

## 5. Limitations

While all of these compounds attempt to target the pathology of MSA, promoting regeneration, especially remyelination, is an underrated therapeutic approach in MSA. In general, remyelination is a physiological repair mechanism in the CNS which decreases over time and is limited to specific regions e.g., inflammatory lesion border in MS [151]. In MSA, myelin formation is strongly affected by α-syn accumulation in oligodendrocytes resulting in myelin loss and consequently in axonal and neuronal loss [13,15,17,19]. Thus, enhancing oligodendroglial differentiation and remyelination in demyelinating diseases may diminish neuronal dysfunction or even slow down disease progression. In the context of MS some efforts have been undertaken to explore new remyelinating therapeutic approaches. Currently, several remyelinating molecules are being tested in clinical trials such as the antihistamines clemastine and GSK239512, the antipsychotic quetiapine, as well as biotin and the antidopaminergic compound domperidone [152,153]. First preclinical studies in a MSA mouse model overexpressing human α-syn under the MBP promoter (line 29) demonstrated, that remyelinating molecules like the antimuscarinic benztropine are able to diminish the myelin deficit and consequently prevent neuronal cell loss in the motor cortex [12].

## 6. Conclusions

In order to accelerate drug development, more sophisticated preclinical MSA models are urgently needed to close the translational gap between preclinical studies and the outcoming of clinical studies in MSA patients. Besides distinct cell lines, primary rodent mixed glial cultures, toxin and genetic animal models, induced pluripotent stem cells derived from MSA patients may be a powerful tool to validate findings from animal models and predict the response of specific molecules in patient-derived neuronal and glial cells [145]. Further considerations should also include combined approaches by targeting different MSA-specific aspects like α-syn pathology and neuroinflammation [90,146].

The fast disease progression and the limited symptomatic treatment result in a very severe impact on the quality of life in MSA patients. Within the last years distinct therapeutic approaches for MSA have emerged and been investigated, however, the majority failed. Nevertheless, numerous novel symptomatic and, more importantly, promising disease-modifying compounds are in the clinical pipeline with the ultimate goal to delay or stop this devastating form of synucleinopathy.

## Figures and Tables

**Table 1:** Overview of current symptomatic treatment strategies in clinical trials for multiple system atrophy (MSA) (status 02/2020, non-comprehensive).

Symptoms	Drug Therapy	Clinical Trials		
		Phase	NCT Number	Ref.
**Parkinsonism**	MAO-B inhibitor safinamide	2	NCT03753763	[50]
**Cerebellar ataxia**	NMDA receptor modulator Tllsh2910	3	NCT03901638	[51]
**Orthostatic hypotension**	α1-receptor inhibitor midodrine	1	NCT02897063	[52]
	Norepinephrine prodrug droxidopa (l-threo DOPS)	1	NCT02897063	[52]
		4	NCT02586623	[53]
	Selective NET inhibitor atomexetine	2	NCT02796209	[54]
	NRI ampreloxetine (TD-9855)	2	NCT03750552	[55]

Monoaminooxidase B (MAO-B), norepinephrine transporter (NET), *N*-methyl-d- aspartic acid (NMDA), norepinephrine reuptake inhibitor (NRI), Reference (ref).

**Table 2:** Drug targets and current clinical trials in MSA (status 02/2020, non-comprehensive).

Therapeutic Targets	Drug Therapy	Clinical Trials		
		Phase	NCT Number	Ref.
**α-Synuclein aggregation**	Vaccines Affitope PD01A and PD03A	1	NCT02270489	[77]
	Epigallocatechin gallate (EGCG)	3	NCT02008721	[78]
	Chelator and antioxidant NMBI (Irminix)	2	NCT04184063	[79]
**Neuroinflammation**	MPO inhibitor verdiperstat	3	NCT03952806	[80]
	CD20-antibody rituximab	2	NCT04004819	[81]
**Neuronal loss**	Bone marrow-derived mesenchymal stem cells (MSCs)	1	NCT03265444	[82]
		1	NCT02315027	[83]
		n.a.	NCT02795052	[84]
		1	NCT04165486	[85]
	Intranasal insulin	2	NCT02064166	[86]
	mTOR inhibitor sirolimus	2	NCT03589976	[87]
	Antioxidant inosine 5′-monophosphate	2	NCT03403309	[88]

Mechanistic target of rapamycin (mTOR), myeloperoxidase (MPO), not applicable (n.a.), and reference (ref).

## Abbreviations

3-NP	3-nitropropionic acid
AD	Alzheimer’s disease
BDNF	brain-derived neurotrophic factor
CGI-C	Clinical Global Impression of Change
CNP	2′-3′-cyclic nucleotide 3′-phosphodiesterase
CNP-hα-syn mice	transgenic MSA mouse model overexpressing α-syn under a 2′-3′-cyclic nucleotide 3′-phosphodiesterase promoter
CNS	central nervous system
EAAT2	excitatory amino acid transporter 2
EGCG	epigallocatechin gallate
GCI	glial cytoplasmic inclusion
GDH2	glutamate dehydrogenase
GDNF	glial cell line-derived neurotrophic factor
hA53T α-syn	human A53T mutant form of α-syn
IL	interleukins
LRRK2	leucine rich repeat kinase 2
MAO-B	monoaminooxidase B
MBP	myelin basic protein
MBP1-hα-syn mice	transgenic MSA mouse model expressing human α-syn under control of the myelin basic protein line 1
MPO	myeloperoxidase
MS	multiple sclerosis
MSA	multiple system atrophy
MSA-C	cerebellar subtype of MSA
MSA-P	parkinsonian subtype of MSA
MSA-QoL	MSA-Quality of Life
MSCs	mesenchymal stem cells
mTOR	mechanistic target of rapamycin
n.a.	not applicable
NET	norepinephrine transporter
NRF2	nuclear-related factor 2
NRI	norepinephrine reuptake inhibitor
NMDA	*N*-methyl-d- aspartic acid
PD	Parkinson’s disease
PDGFβ	platelet-derived growth factor β
PLP-hα-syn mice	transgenic MSA mouse model overexpressing human α-syn under the proteolipid protein promoter
PSP	progressive supranuclear palsy
TNFα	tumor necrosis factor alpha
TPPP/p25a	tubulin polymerization-promoting protein
UMSARS	Unified Multiple System Atrophy Rating Scale
VEGF	vascular endothelial growth factor
α-syn	alpha-synuclein
